# Costs of cold acclimation on survival and reproductive behavior in *Drosophila melanogaster*

**DOI:** 10.1371/journal.pone.0197822

**Published:** 2018-05-23

**Authors:** Elizabeth R. Everman, Jennifer L. Delzeit, F. Kate Hunter, Jennifer M. Gleason, Theodore J. Morgan

**Affiliations:** 1 Division of Biology, Kansas State University, Manhattan, Kansas, United States of America; 2 Department of Ecology and Evolutionary Biology, University of Kansas, Lawrence, Kansas, United States of America; Lancaster University, UNITED KINGDOM

## Abstract

Fitness is determined by the ability of an organism to both survive and reproduce; however, the mechanisms that lead to increased survival may not have the same effect on reproductive success. We used nineteen natural *Drosophila melanogaster* genotypes from the *Drosophila* Genetic Reference Panel to determine if adaptive plasticity following short-term acclimation through rapid cold-hardening (RCH) affects mating behavior and mating success. We confirmed that exposure to the acclimation temperature is beneficial to survival following cold stress; however, we found that this same acclimation temperature exposure led to less efficient male courtship and a significant decrease in the likelihood of mating. Cold tolerance and the capacity to respond plastically to cold stress were not correlated with mating behavior following acclimation, suggesting that the genetic control of the physiological effects of the cold temperature exposure likely differ between survival and behavioral responses. We also tested whether the exposure of males to the acclimation temperature influenced courtship song. This exposure again significantly increased courtship duration; however, courtship song was unchanged. These results illustrate costs of short-term acclimation on survival and reproductive components of fitness and demonstrate the pronounced effect that short-term thermal environment shifts can have on reproductive success.

## Introduction

Variation in temperature can negatively impact the survival and reproduction of ectothermic organisms as a result of their sensitivity to small changes in temperature [1–4]. Diverse mechanisms, including physiological tolerance, phenotypic plasticity, and modification of behavior, allow for the maintenance of survival and reproductive fitness in response to environmental stress [4–11]. Of these mechanisms, both basal thermal tolerance and phenotypic plasticity can overcome the negative fitness impacts of thermal variation. Phenotypic plasticity in variable thermal environments generally leads to survival benefits [1,12–14]; however, the influence of the temperature fluctuation on other components of fitness has received less attention [15]. A broad understanding of the effects of thermal variation on multiple components of fitness can provide insight into the persistence and evolutionary trajectory of populations.

One process that can provide a survival benefit through phenotypic plasticity in ecotherms is rapid cold-hardening (RCH) [13]. RCH is a type of short-term acclimation that results in increased cold tolerance over very brief periods of time (minutes to hours) [1–3,7,16–22]. In nature, temperature can rapidly shift from warm to cool over several hours as a result of diurnal variation in temperature. On occasion, these fluctuations are extreme, as in the case of fast-moving weather systems and increased diurnal variability associated with sesonal transitions [23–27]. RCH has been shown to increase survival in response to natural shifts in temperature [1–3,7,16–22]. For example, Koveos (2001)[19] demonstrated in a field experiment with natural conditions that exposure of *Bactrocera oleae* to diurnal temperature shifts in early spring from approximately 10°C to approximately -2°C significantly increased survival of flies at -7°C compared to flies that had not experienced any temperature fluctuation. Similarly, exposure of *Drosophila melanogaster* to natural temperature shifts in a field experiment also improved cold survival and decreased chill-coma recovery time compared to flies that did not experience temperature fluctuation [1]. These shifts in cold tolerance under natural field conditions suggest that cold tolerance is highly sensitive to diurnal fluctuations in temperature and that RCH has an ecologically important role in increasing survival under natural conditions.

The increased survival of indiviuals following RCH under natural conditions suggests that the RCH response is adaptive [28]. In addition to field studies mentioned above, the benefits of RCH on insect cold tolerance have been repeatedly illustrated in the laboratory by demonstrating increased survival, decreased chill-coma recovery time, decreased supercooling point, and critical thermal minimum in individuals following a rapid shift in temperature to a mild acclimation treatment temperature [1–3,7,12,16,17,20–22,29–32]. In the laboratory, short-term temperature fluctuations that induce RCH in nature are modeled by exposing individuals to gradual or abrupt changes in temperature that approximate natural conditions [1,2,4,19]. The high correspondence between laboratory and field studies suggests that simple lab assays provide insight into how natural populations respond to temperature variability in general [1,2,17].

Although RCH has beneficial effects on physiological metrics of cold tolerance, this response is also genetically variable, and not all genotypes benefit from RCH [12,31,33]. Further, the response to acclimation through RCH, quantified as RCH capacity, varies across environments [18,32], and benefits from RCH for survival do not necessarily imply uniform benefits of RCH for other components of fitness [4,12,34]. For example, exposure to non-lethal temperature has been shown to have a mix of positive and negative effects on reproductive fitness in a diverse group species [5,10,15,28,34–38]. Thus, individuals that have increased survival induced by RCH (or positive RCH capacity) may experience lower reproductive success or negative effects on other components of fitness from the same exposure to the acclimation temperature [28,34,37]. At the same time, genotype-specific capacity for plastic response to temperature stress (i.e. genetic variation in phenotypic plasticity) may influence the effect of temperature on reproductive success. Understanding the relationship between physiological and behavioral responses to RCH on both survival and reproduction in distinct genotypes can provide insight into the costs for maintaining phenotypic plasticity for overall fitness.

*Drosophila melanogaster* is excellent for modeling the influence of temperature on mating behavior because males have predictable, complex courtship behaviors that are sensitive to environmental variation. During male courtship, the male follows the female, orients near the female’s head, and produces courtship song by vibrating a wing extended perpendicular to his body [39,40]. These wing movements produce an audible, species-specific song, which in *D*. *melanogaster* consists of pulse and sine elements that are known to influence female receptivity (S1 Fig) [41–43].

Temperature influences pulse song and other aspects of male courtship behavior in many drosophilid species, both at the time of courtship and as a result of acclimation to seasonal and short-term fluctuations in temperature [4,15,35,36,44–46]. Seasonal acclimation to stressful temperatures in male *D*. *montana* and *D*. *littoralis* can result in decreased mating success [44,45]: males collected during warm months produce higher frequency pulse song and have higher mating success than overwintered males, which is linked to female preference for high frequency pulse song in this species [45]. Short-term temperature stress can have a similar negative influence on mating success in *Drosophila melanogaster* and other invertebrates [4,10]. For instance, a change in temperature of 7°C negatively affects mating success in *D*. *melanogaster*, but can be mitigated if flies are allowed to acclimate to the same lower temperature prior to the experiment [4]; thus, a plastic behavioral response to the thermal environment can benefit reproduction. This implies that a larger temperature shift, such as that experienced through acclimation prior to harsh cold stress, will have a significant effect on mating behavior.

In this study, we addressed several open questions by assessing the costs and benefits of RCH on survival and reproductive components of fitness. Overall, we expected short-term thermal acclimation through RCH to have a positive effect on survivorship following cold stress relative to individuals that experienced cold stress at -6°C and to have a negative effect on mating behavior and success relative to individuals maintained at 25°C. Because levels of basal cold tolerance, RCH capacity, and mating behavior are genetically controlled [12,47], we also expected that genotype-specific levels of temperature tolerance would interact with the negative impact of exposure to acclimation temperature on mating success.

We tested these genotypic effects by measuring thermal tolerance, plasticity, and reproductive behavior in nineteen unique, naturally derived *D*. *melanogaster* genotypes from the *Drosophila* Genetic Reference Panel (DGRP). The DGRP consists of multiple inbred genotypes and provides a powerful tool for measuring identical genotypes across environments and traits. This panel was recently used to characterize the genetic control of RCH capacity as well as variation in mating behavior, establishing a genetic basis for each trait [12,47]. We used genotypes from the DGRP to investigate how the genetic variability in plasticity and mating are related following acclimation and non-acclimation exposure. We also tested potential mechanisms by which exposure to acclimation temperature influenced mating success by examining the effect of acclimation on specific components of mating behavior. Because previous research has shown that courtship song is altered by temperature, we expect specific elements of courtship behavior, including pulse song, to be negatively impacted by exposure to the short-term thermal acclimation temperature that leads to the RCH survival response.

## Materials and methods

### Fly stocks

The DGRP is a collection of 205 inbred and fully-sequenced *D*. *melanogaster* lines established by sampling naturally segregating genetic variation in a single population in Raleigh, North Carolina [48,49]. Nineteen lines were selected from the DGRP that have been previously shown to vary in cold tolerance and RCH capacity [31,48]. We chose a subset of nineteen lines from the DGRP to perform detailed analyses of courtship behavior and song (discussed below). All flies were reared on standard cornmeal/molasses/agar media at 25 ± 1°C in narrow polystyrene vials (25 x 95mm) on a 12:12 hour light:dark cycle.

All experimental flies were reared at moderate larval density by placing five males and five females in each vial and allowing females to lay eggs for three days. After three days, the parents were transferred to new vials. In the new vials, females continued oviposition for another three days before all parents were discarded. Experimental flies were obtained from both sets of vials. Non-virgin individuals were collected for use in cold tolerance assays [12,50]. At zero to two days of age, flies were sorted over light CO_2_ and placed in groups of 10 same-sex individuals per vial. Flies were tested at five to seven days of age to measure cold tolerance. For mating experiments, virgin males and females were collected from each DGRP line and were sorted over light CO_2_ within four hours of eclosion and maintained as individuals in vials. Virgin flies were used in mating and song assays when seven days old.

All response variables were assessed for normality and homoscedasticity via the Shapiro Wilk test and the Levene test, respectively. All variables recorded in this study deviated from the assumptions of parametric tests, and the issues with normality remained following data transformation. Because parametric tests are generally robust to violations of normality and homoscedasticity [51], we proceeded with parametric tests of our data, as many models were too complicated to be tested non-parametrically, and type I error is prone to inflation when the number of observations are high in non-parametric tests [52,53]. As a result, our analyses are conservative assessments (relative to nonparametric testing) of the influence of genotype and treatment on survival and behavior. All analyses were performed in R [54–56] unless otherwise indicated. All data are available in S1 File.

### Cold tolerance assay and analysis

**Fig 1:**
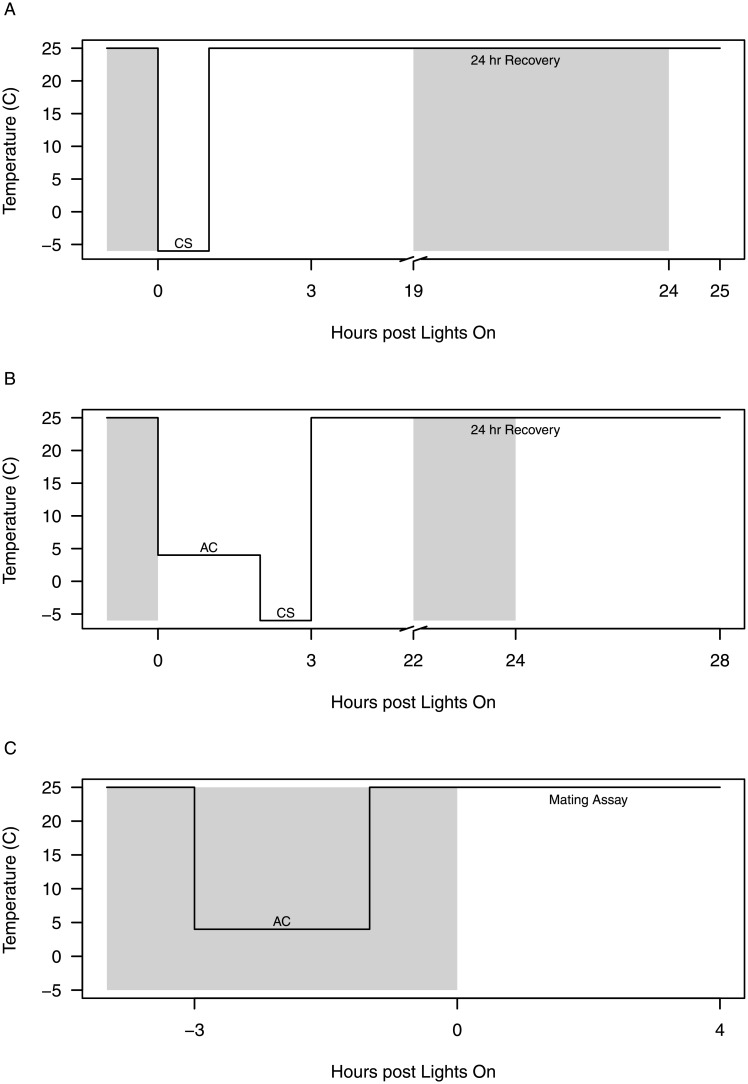
Temperature treatments for the cold tolerance and mating assays. Flies were exposed to either (A) a basal cold tolerance treatment for 1 hour at -6°C or (B) an acclimation treatment for two hours at 4°C to induce RCH followed by -6°C for 1 hour to determine the level of cold tolerance for each genotype. C. Flies were exposed to either the acclimation treatment temperature (4°C) for 2 hours to induce RCH or were held at 25°C for the mating latency and song assays. Shading indicates the timing of lights on for experimental flies used in each assay.

To characterize the response to cold stress for each isogenic line, males and females of each DGRP line were separated into two groups. We chose our temperatures following Gerken *et al*. (2015) [12]. To measure basal cold tolerance, vials containing 10 individuals from each line and sex were exposed to -6°C for one hour (Fig 1A). After a recovery period of 24 hours at 25°C, the percent survival of each vial was calculated. To measure acclimated cold tolerance, vials containing 10 individuals from each line and sex received a mild cold stress at 4°C for two hours before they were transferred to -6°C for an hour (Fig 1B). After a recovery period of 24 hours at 25°C, percent survival of each vial was calculated. Measures of basal and acclimated cold tolerance were replicated four times for each sex in each of the nineteen lines.

The effects of acclimation and DGRP line on the proportion of flies that survived cold stress at -6°C were tested with a three-way mixed-model ANOVA, where line was a random effect and sex and acclimation treatment were fixed effects. Rapid cold-hardening (RCH) capacity was calculated as the difference between the mean proportion of flies that survived the acclimation cold tolerance assay and the mean proportion that survived the basal cold tolerance assay [12].

### Mating latency assay and analysis

To investigate the effect of exposure to the acclimation temperature for inducing RCH on reproductive fitness, we quantified two metrics of *D*. *melanogaster* mating behavior in groups of flies that were either exposed to the acclimation temperature or maintained at 25°C. All of the DGRP lines were measured on each experimental day. Three hours prior to lights on, seven-day-old virgin flies in the acclimation treatment were transferred to 4°C for two hours (Fig 1C). These flies were then transferred to fresh media and allowed to recover from acclimation temperature-induced coma for an hour at 25°C. Control flies were maintained at 25°C prior to the mating assay. At lights on, male and female flies from each treatment were paired within and between the acclimation and control treatments (i.e., acclimated male x acclimated female; control male x acclimated female; acclimated male x control female; control male x control female) for each DGRP line. Each pair was observed for four hours at 25°C in vials containing media and activated yeast because the effects of acclimation have been demonstrated to last between four and eight hours in *D*. *melanogaster* [3,31].

During the four-hour screen, we measured courtship latency (the time until males started courtship behavior) and courtship duration (the time from the start of courtship to the time when the male successfully began copulation). Males that did not engage in courtship were given a courtship latency of 14,400 seconds (4 hours) and included in the courtship latency analysis, because every pair eventually mated and produced offspring when maintained as pairs for the week following the screen. Males that did not mate within the four-hour screen were excluded from the courtship duration analysis. Data were collected with five to eight replicates per line and treatment combination (S1 Table). Two three-way mixed-model ANOVAs were used to test the effect of acclimation treatment and DGRP line on courtship latency and courtship duration. In both analyses, line was a random effect and the sex-specific acclimation treatments were fixed effects. The effect of acclimation on the probability of mating was analyzed with a chi-square test.

We used regression analysis to determine the effect of genotype-specific levels of basal cold tolerance and RCH capacity on courtship latency and courtship duration. Because mixed model ANOVA results indicated that only the mating behavior of males was negatively impacted by the acclimation treatment, we used male-specific average RCH capacity and basal cold tolerance for each DGRP line and compared these responses to courtship latency and courtship duration for the control pair of flies and for the pair in which only the male was exposed to the acclimation temperature. Behavioral plasticity following acclimation temperature exposure was calculated by subtracting the average courtship duration of acclimated males from the average courtship duration of control males. As in the previous analysis, we only included the pairs with control females. We analyzed these data using linear regression to test the relationship between behavioral and physiological plasticity measured as RCH capacity.

### Courtship song assay and analysis

To examine costs of acclimation on courtship in lines that have an adaptive survival response to the treatment, we selected the five lines with the highest positive RCH capacities that also were among those possessing the most negative male behavioral plasticity scores (RAL-362, RAL-517, RAL-365, RAL-153, RAL-195). These lines were measured for the effect of the acclimation temperature on courtship song. We used a subset of the original nineteen lines because collection of song data was labor intensive. All lines were measured on each experimental day.

Experimental flies for the courtship song assay were reared at 25°C, collected as virgins over CO_2_ within four hours of eclosion, maintained in groups of five same-sex individuals, and were tested at seven days of age. Males were divided between the acclimation temperature exposure and control treatments; the acclimation temperature exposure treatment was the same as above for the mating latency experiments. Females were maintained at 25°C because the primary effect of acclimation temperature exposure on mating behavior was in the males; therefore, females were standardized to determine the effect of exposure to the acclimation temperature on male song. Over the five DGRP lines, 15.6 ± 2.70 S.D. males per line received the acclimation treatment and 14.2 ± 2.05 S.D. males per line received the control treatment (S1 Table).

To record song, a virgin male and a virgin female were paired in a clear circular mating chamber (25 mm diameter, 12 mm height), with a mesh bottom. The mating chamber was placed directly over an Insectavox-style microphone [57] modified for simultaneous video capture. The microphone was an Electret noise-cancelling microphone (PUI Audio, Inc. ANM-5254L-R) with an SSM2019 audio Preamp (Analog Devices). Video was captured with a standard video microscope with 800X variable magnification. Recording was captured with a video capture system (FC500, Diamond) and stored on a computer. Recording continued until either copulation began or until fifteen minutes had elapsed if the pair did not copulate. Because we had only one microphone, recordings of pairs could only be done one at a time. Recordings were made up to four hours post lights on each day. The order of DGRP lines and treatment of the males was randomized each recording day. Ambient temperature was recorded at the start and end of the recording window. Recording temperature was the mean of the starting and ending temperature. The difference in temperature between the start and end of each recording was on average 0.0856°C ± 0.13 S.D., and the median difference was 0.1°C.

To measure the song characters, MP4 video files were converted to MP3 audio files with MacX Free MP3 Video Converter Version 4.1.8 (Digitary Software). MP3 sound files were filtered with a high pass frequency of 100 Hz and a low pass frequency of 1000 Hz and saved as WAV files in Audacity^®^ 2.1.1. Pulses were identified on oscillograms and the time between pulses, the interpulse interval (IPI), was measured manually in all bursts of at least three pulses (S1 Fig). Because IPI varies with temperature, the slope of a regression analysis of the mean IPI of all songs against recording temperature (-1.0437) was used to correct all IPI data to a common temperature (25°C) using the equation

correctedIPI=−1.0437(25°C−recordingtemperature)+measuredIPI.

We used bootstrapping to determine the minimum number of IPI needed to accurately estimate the mean IPI for each song (R code is provided in S2 File). To identify the minimum number of IPI needed per song to estimate mean IPI, we selected the eight songs with the most IPI per song, equivalent to 5% of the 148 recorded songs. We then randomly sampled 3, 5, 10, 15, 20, 25, and 30 IPI from each song with 10,000 iterations. The mean IPI of each iteration was used to calculate the overall mean IPI, standard deviation, and 95% confidence interval for each IPI sample size for each of the eight songs. The distribution of mean IPI for each IPI sample and song was then compared to the mean IPI calculated from the entire song. Visual examination of the standard deviation and 95% confidence intervals of the bootstrapped means for each IPI sample size and song demonstrated that the variation around the bootstrapped means stablized as the number of IPI sampled reached 15 IPI (S2 Fig). The estimation of the mean IPI per song was not greatly improved when more than 15 IPI were used (S2 Fig). For subsequent analyses, mean IPI was only calculated for songs with at least 15 IPI resulting in a total of 74 songs analyzed.

From the videos, we recorded mating success and calculated courtship duration, courtship index (the proportion of the courtship duration in which the male was actively courting), and song index (the proportion of the courtship duration in which the male was actively singing) for mated and unmated males. Song index was determined by adding together all IPIs for a male to calculate the total time spent singing and dividing that number by the male’s courtship duration. Both song parameters and courtship parameters were scored blindly with respect to line and treatment. Males that did not court or sing were not included in these calculated variables.

To assess the broad effect of exposure to the acclimation temperature on courtship behavior and likelihood of mating, we tested the effect of male treatment on mating success, courtship occurrence, and song occurrence with chi-square tests. We then tested the effect of DGRP line, male treatment, and mating status (whether males mated or not) on mean IPI, courtship index, song index, and courtship duration individually with mixed-model three-way ANOVAs. Sample sizes are presented in S1 Table.

## Results

### The effect of acclimation and genetic variation on survivorship

**Fig 2:**
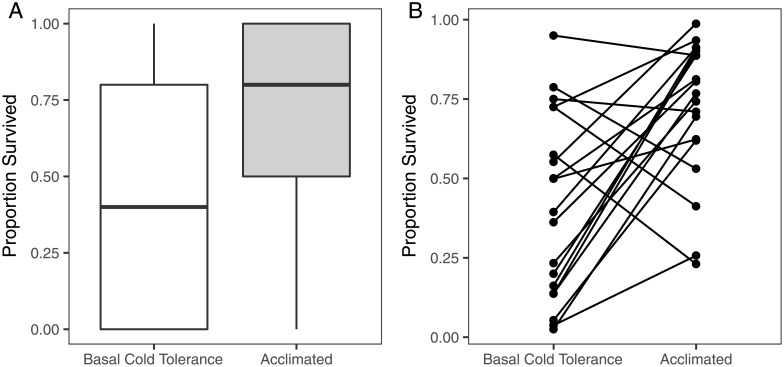
Survival following cold stress with and without the acclimation pre-treatment. A. Flies that were acclimated had higher survival than flies that did not receive the acclimation treatment prior to cold stress. B. Genotype significantly influenced survival following the basal cold tolerance and acclimation treatments. Each point and connecting line represents the change in a genotype’s average survival between the basal cold tolerance and acclimation treatments. This change (acclimation survival—basal survival) is RCH capacity, one measure of phenotypic plasticity. Variation in change in survival between the two treatments led to genotype-specific variation in RCH capacity.

Survivorship following exposure to cold stress was influenced both by treatment and genetic variation. The acclimation treatment significantly improved survivorship compared to the basal cold tolerance treatment, indicating most individuals had positive RCH capacity (*T* = -3.8, *P* < 0.001; Table 1; Fig 2A). Treatment and DGRP line interacted to influence survivorship following the acclimation and basal cold tolerance treatments as well, indicating that the genotypes included in this study responded differentially to the cold stress treatments (Fig 2B; χ^2^ = 21.7, *P* < 0.001); thus, the DGRP lines had different levels of cold tolerance and RCH capacity. Genotype-specific responses to cold with acclimation, without acclimation, and RCH capacity point to genetically determined differences in the physiological response to cold stress. The lack of a sex-specific response suggests that male and female survival was influenced by treatment in a similar manner (*T* = -0.42, *P* = 0.67).

**Table 1:** Mixed effects ANOVA of survival and mating behavior with and without acclimation in nineteen DGRP lines.

Response	Source	df	Estimate (± S.E.)	T value	P value
**Survival following cold stress**	Sex	37.8	-0.0200 (0.05)	-0.424	0.67
	TRT*	25.5	-0.337 (0.09)	-3.83	**< 0.001**
	Sex x TRT	37.8	0.0667 (0.07)	0.932	0.36
**Courtship Latency**	Male TRT	48.5	344 (584)	0.589	0.56
	Female TRT	57.9	60.3 (528)	0.114	0.91
	Male x Female TRT	1443	7.94 (703)	0.011	0.99
**Courtship Duration**	Male TRT	525	1775 (566)	3.14	**< 0.01**
	Female TRT	51.3	245 (560)	0.436	0.66
	Male x Female TRT	784	-1293 (816)	-1.59	0.11

*TRT = Treatment; significant results are in bold

### The effect of acclimation and genetic variation on mating behavior

**Fig 3:**
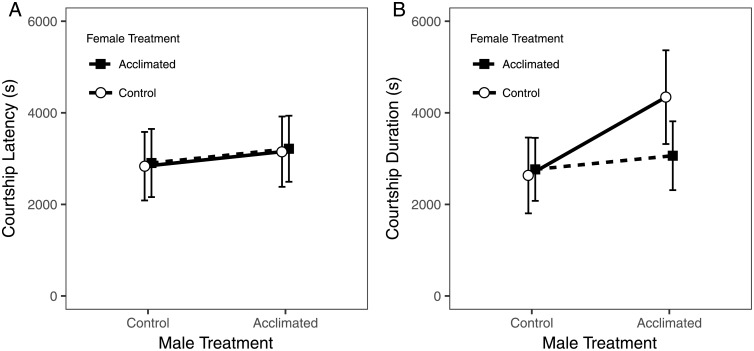
Courtship latency and courtship duration with and without acclimation. A. Exposure to the acclimation treatment did not impact courtship latency when either the male or female received the acclimation treatment. B. Males exposed to the acclimation temperature had significantly longer courtship duration when paired with control females. Means are shown with 95% confidence intervals.

The exposure of males and females to the cold acclimation temperature did not increase courtship latency (male treatment: *T* = 0.59, *P* = 0.56, female treatment: *T* = 0.11, *P* = 0.91, male by female treatment interaction: *T* = 0.011, *P* = 1; Fig 3A, Table 1). However, exposure of males to the cold acclimation temperature significantly increased courtship duration, indicating that exposure to the acclimation temperature decreased the efficiency of courtship (*T* = 3.14, *P* < 0. 01; Fig 3B, Table 1). Chi square analysis of the proportion of pairs that mated found that males that received the acclimation treatment were significantly less likely to mate than control males (*χ*^*2*^ = 20.2, *P* < 0.0001; data not shown). The acclimation temperature exposure of females did not influence courtship duration (*T* = 0.44, *P* = 0.66; Table 1). Courtship duration was also not influenced by an interaction between the male and female acclimation temperature treatment (*T* = -1.59, *P* = 0.11; Table 1), indicating that the negative effect of cold acclimation on courtship duration was driven by the ability of males to court and not by the receptivity of females. The random effects included in the mixed model ANOVA DGRP line and the interaction between DGRP line and either male or female treatments did not significantly influence either behavioral response measured (courtship latency: *χ*^*2*^ = 0, *P* = 1; courtship duration: *χ*^*2*^ = 0, *P* = 1; S3 Fig). Collectively, these results show that the cold acclimation temperature exposure negatively affected mating as measured by courtship duration.

To determine if genetic variation in the physiological response to acclimation and cold stress was correlated with the behavioral response to the acclimation temperature, we calculated the correlation between DGRP line-specific mating behavior variables (courtship latency and courtship duration) and male basal cold tolerance and RCH capacity. Under control mating conditions (neither the male nor the female received the acclimation treatment) and when only the male received the acclimation stress, neither basal cold tolerance (BCT) nor RCH capacity was correlated with courtship latency (Control pair: RCH: *F*_*1*,*17*_ = 0.07, *P* = 0.8, R^2^ = 0.004; BCT: *F*_*1*,*17*_ = 1.8, *P* = 0.2, R^2^ = 0.1; Stressed male: RCH: *F*_*1*,*17*_ = 1.1, *P* = 0.3, R^2^ = 0.06; BCT: *F*_*1*,*17*_ = 0.2, *P* = 0.7, R^2^ = 0.01). RCH capacity was weakly negatively correlated with control male courtship duration (*F*_*1*,*17*_ = 3.9, *P* = 0.06, *R*^*2*^ = 0.2); however, when males were exposed to the acclimation temperature, the relationship was much weaker (*F*_*1*,*17*_ = 0.5, *P* = 0.5, *R*^*2*^ = 0.03). Basal cold tolerance was positively correlated with control male courtship duration (*F*_*1*,*17*_ = 4.9, *P* < 0.05, *R*^*2*^ = 0.2), but as with RCH capacity, the relationship was not significant when males received the acclimation stress (*F*_*1*,*17*_ = 0, *P* = 1, *R*^*2*^ = 0.0).

The change in courtship behavior between the control and acclimation temperature treatment is a measure of behavioral plasticity within a genotype. To further investigate the influence of RCH capacity on the change in courtship duration when males receive the acclimation treatment, we measured the correlation between RCH capacity and behavioral plasticity. This approach allows us to determine if the capacity for a physiological response relates to the behavioral response to the acclimation temperature. We found no relationship between behavioral plasticity in courtship duration and RCH capacity (physiological plasticity) (data not shown, *F*_*1*,*17*_ = 0.18, *P* = 0.67). Given the marginal effects of genetic variation in basal cold tolerance and RCH capacity on mating behavior, we conclude that genetic variation in the acclimation response has little influence on courtship latency and courtship duration when males experienced the acclimation temperature.

### The effect of acclimation temperature on male courtship song

**Fig 4:**
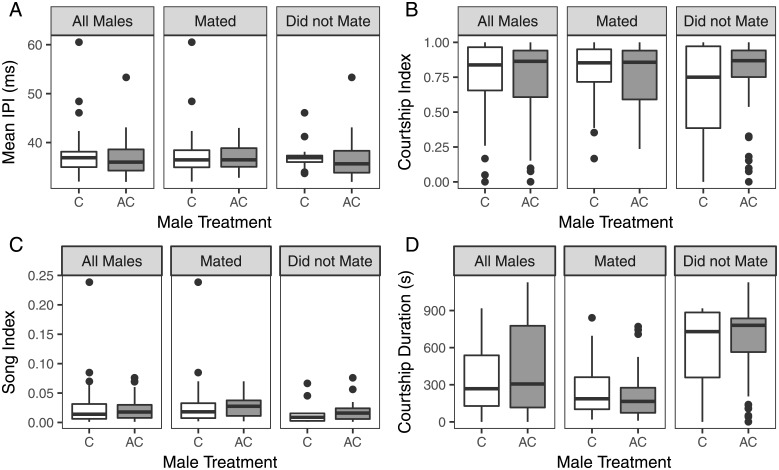
Effects of exposure of males to acclimation temperature on courtship parameters measured in five genotypes. Boxplots show males that were exposed to the acclimation temperature (AC) were not significantly different from control (C) males when mean IPI (A: *F*_*1*,*73*_ = 0.73, *P* = 0.40), courtship index (B: *F*_*1*,*128*_ = 0.0015, *P* = 0.97), or song index (C: *F*_*1*,*73*_ = 0.21, *P* = 0.65) was compared. D. Males exposed to the acclimation temperature had slightly longer courtship duration compared to control males (*F*_*1*,*128*_ = 3.89, *P* = 0.05).

Because the five genotypes used in the courtship song assay had high positive RCH capacities and similar negative behavioral responses to the acclimation temperature treatment (Tukey’s HSD; all comparisons *P* > 0.05), we did not analyze each genotype separately. As with the mating latency assay, the male acclimation temperature treatment had a significant negative effect on mating success (*χ*^*2*^ = 6.92, *P* < 0.01): 72.5% of the control males successfully copulated, whereas only 51.3% of the acclimation temperature exposed males mated within the fifteen-minute assay period. Exposure to the acclimation temperature did not influence courtship occurrence as all but two males across the experiment engaged in courtship behavior (*χ*^*2*^ = 0.4, *P* = 0.53). Of the males that courted, 83.3% of the acclimated temperature exposed males and 78.6% of the control males produced pulse song (*χ*^*2*^ = 0.28, *P* = 0.60). Therefore, the negative effect of the acclimation temperature treatment on mating success was not the result of an inability of acclimated males to court or sing. Exposure of males to the acclimation temperature did not influence mean IPI of the entire song (*F*_*1*,*73*_ = 0.73, *P* = 0.40; Table 2, Fig 4A), courtship index (*F*_*1*,*128*_ = 0.0015, *P* = 0.97; Table 2, Fig 4B), or song index (*F*_*1*,*73*_ = 0.21, *P* = 0.65; Table 2, Fig 4C). However, males exposed to the acclimation temperature did have marginally longer courtship durations (*F*_*1*,*128*_ = 3.89, *P* = 0.05; Table 2, Fig 4D).

**Table 2:** ANOVA of song recording variables with and without acclimation in five DGRP lines.

Response	Source	df	MS	F value	P value
**Mean IPI**	Male TRT*	1	11.6	0.729	0.40
	Line	4	98.3	6.21	**< 0.001**
	Mated vs. Not	1	2.13	0.135	0.71
	Male TRT x Line	4	11.9	0.751	0.56
	Male TRT x Mated vs. Not	1	6.87	0.433	0.51
	Line x Mated vs. Not	4	1.95	0.123	0.97
	Male TRT x Line x Mated vs. Not	3	15.4	0.973	0.41
	Residuals	73	15.8		
**Courtship Index**	Male TRT	1	9.00 x 10^−5^	0.0015	0.97
	Line	4	0.0825	1.33	0.26
	Mated vs. Not	1	0.0701	1.13	0.29
	Male TRT x Line	4	0.122	1.97	0.10
	Male TRT x Mated vs. Not	1	0.0434	0.702	0.40
	Line x Mated vs. Not	4	0.104	1.68	0.16
	Male TRT x Line x Mated vs. Not	4	0.0258	0.416	0.80
	Residuals	128	0.0619		
**Song Index**	Male TRT	1	1.91 x 10^−4^	0.214	0.65
	Line	4	2.34 x 10^−3^	2.61	**< 0.05**
	Mated vs. Not	1	1.52 x 10^−5^	0.0170	0.9
	Male TRT x Line	4	6.01 x 10^−4^	0.670	0.61
	Male TRT x Mated vs. Not	1	2.10 x 10^−5^	0.0234	0.88
	Line x Mated vs. Not	4	8.25 x 10^−4^	0.920	0.46
	Male TRT x Line x Mated vs. Not	3	4.93 x 10^−4^	0.549	0.65
	Residuals	73	8.97 x 10^−4^		
**Courtship Duration**	Male TRT	1	2.27 x 10^5^	3.89	**0.05**
	Line	4	5.73 x 10^5^	9.83	**< 0.001**
	Mated vs. Not	1	3.01 x 10^6^	51.6	**< 0.001**
	Male TRT x Line	4	6.61 x 10^4^	1.13	0.34
	Male TRT x Mated vs. Not	1	3.19 x 10^4^	0.547	0.46
	Line x Mated vs. Not	4	2.12 x 10^5^	3.64	**< 0.01**
	Male TRT x Line x Mated vs. Not	4	3.87 x 10^4^	0.665	0.62
	Residuals	128	5.83 x 10^4^		

*TRT = Treatment; significant results are in bold

## Discussion

### Cold-acclimation capacity has costs and benefits on survivorship and mating success

The ability of an organism to thrive in a thermally variable environment influences both survival and the reproduction of the organism. In a simple scenario, an organism that survives cold temperature fluctuations can reproduce in the future; in this way, the survival benefit of acclimation also indirectly benefits reproductive success. However, non-lethal temperature exposure can directly alter reproductive success [4,34], and while acclimation can greatly benefit survival, this form of mild cold stress can be very costly for reproductive success. The presence of benefits and costs for survival and reproductive success is clearly demonstrated in our study.

Survival following cold stress without acclimation (basal cold tolerance) was genetically variable, and most lines exposed to the acclimation temperature before cold stress showed a significant increase in survival through RCH (Fig 2B). Positive RCH capacity is consistent with previous reports of a beneficial effect of acclimation through RCH on survival [1–3,7,12,16,17,20–22,29–32]. However, the presence of lines in our study that had negative RCH capacity (lower survival following RCH) suggests that there is a physiological cost of acclimation for some genotypes. This cost may be because of the acclimation treatment alone or the combined effect of acclimation followed by the cold shock treatment [12,31,33]. Under natural conditions, lines with positive RCH capacity would have the greatest likelihood of survival following a temperature shift from warm conditions during the day to colder overnight temperatures. In a similar assay carried out under field conditions, Kelty (2007) demonstrated that cold tolerance (assessed by exposing flies to -6°C for one hour) significantly increased by 6.8 fold after flies were allowed to rapidly-cold harden in the field over a 12°C decline in temperature compared to flies that did not experience RCH. The large increase in survival following RCH acclimation to 4°C observed in our study, as well as other studies carried out in the laboratory and field, support the interpretation that RCH benefits are gained through exposure to mild cool temperatures and are ecologically relevant for natural environmental shifts in temperature [1,7,12,16,17,19–22,29–32,58,59].

Although most DGRP lines had positive RCH capacity, the exposure of males to the acclimation temperature had a significant negative effect on courtship duration and copulation success. The increased courtship duration when males, but not females, were exposed to the acclimation temperature suggests that mild cold exposure resulted in reduced attractiveness of male courtship behavior. This implies that females either selected against or were insufficiently stimulated by males that had experienced a stressful environment (Fig 3B). However, females exposed to the acclimation temperature accepted control and acclimation exposed males in the same amount of time (Fig 3B, Table 1). One interpretation of this pattern is that females may mate assortatively with males that are of equal or better (i.e. less stressed) physiological status. Assortative mating under varying thermal conditions has been demonstrated in *D*. *melanogaster* and other species when populations are maintained at different temperatures over many generations [60–62]. Our data suggest that assortative mating is possible after brief shifts in temperature, but this pattern should be verified through the study of female preference given a choice between stress and non-stressed males. The negative effect of exposure to cold temperature on mating is likely highly relevant for reproductive success under natural conditions as *D*. *melanogaster* males typically court during the early morning hours, when temperatures are increasing from recent overnight low temperatures [63]. The lower reproductive success of acclimated males suggests that under natural conditions, males that go into a cold topor state following exposure to cold temperatures will have a reproductive disadvantage in comparison to males that avoided exposure to cold temperature through use of refugia.

The negative effect of exposure to the acclimation temperature on courtship duration observed in our study is comparable with the decrease in mating success of *D*. *montana* males exposed to chronic winter-like temperatures [45] and *D*. *melanogaster* exposed to temperatures lower than their rearing temperature [4]. Our results demonstrate that fitness effects following exposure to 4°C acclimation are context-dependent, and that courtship behavior in *D*. *melanogaster* is sensitive to short-term temperature fluctuations that can occur over a period of a few hours. Genetic variation in basal cold tolerance and RCH capacity did not have a large influence on the effect of exposure to the acclimation treatment on mating behavior. This may suggest that the effects of cold temperature on survival traits are independent of their effects on behavioral traits. If this is the case, survival and behavioral components of fitness may be able to evolve independently. Further study is necessary to determine if the lack of a genetic correlation between our measures of physiological and behavioral response to cold stress and acclimation is a characteristic particular to our experimental DGRP population or indicative of a more widespread pattern that influences the evolution of natural, outbred populations. Although we used a simple assay of cold acclimation with rapid temperature shifts to model short-term thermal variation, our findings point to an interesting relationship between thermal stress and genetic variation in cold tolerance, RCH capacity, and reproductive behavior that can be used to inform future studies using fluctuating temperatures that replicate natural conditions.

### Decreased mating success following exposure to acclimation temperature is not caused by changes in courtship song

To further explore the negative effect of exposure to the acclimation temperature demonstrated by males (Fig 3B), we examined courtship song. We expected mean IPI to be influenced by acclimation, given the previous report of temperature-induced changes to pulse song in overwintered *D*. *montana* males [45]. However, in our experiment, exposure to acclimation temperature did not affect mean IPI, courtship index, or song index. These variables, all related to specific elements of male courtship, did not provide a mechanistic explanation for the negative effect of acclimation on mating success (Fig 4A–4C).

IPI is one of many mating signals that may contribute to male attractiveness during courtship. Other song parameters, including IPI cycles [64,65] may play a role in mating success, though temperature dependency in *D*. *melanogaster* has not been examined. Other mating signals that are affected by temperature include cuticular hydrocarbons (CHCs) that act as pheromones, which are important for female response to males [66–70]. Although developmental temperature can change CHC composition [67–70], little is known about the effect of short-term temperature fluctuations (as tested here) on CHCs. If flies are exposed to short-term temperatures that are distinct from rearing conditions, the composition of CHCs in *D*. *melanogaster* may be changed [65]; however, additional research is necessary to determine how very brief exposures to stressful cold temperatures (such as the acclimation treatment used in this study) alters the CHC composition of males.

Further testing is required to identify the mechanism through which acclimation impacts mating success in *D*. *melanogaster* males. Measuring the physiological limits of individuals under multiple conditions is difficult, as is correlating physiological response of outbred genotypes to behavioral response. Our use of inbred lines from the DGRP allowed us to assess genotype-specific patterns in behavior and physiology across multiple environments with replicated measures of the same genotype. Although the lines are inbred, the DGRP is an excellent tool for investigating the relationship between genetically controlled phenotypes in the context of natural genetic variation. Because the lines are derived from a natural population, they represent a snapshot of standing genetic variation in a population and allow examination of the effects of recessive polymorphisms that influence phenotypic responses. While the inbred nature of each DGRP line could alter male courtship behavior independently of the acclimation treatment, the use of these naturally derived lines provides insight into how genetic variation in physiology and behavior interacts with short-term environmental variation to determine whole organism fitness.

### Conclusions

We addressed several significant aspects of the effect of exposure to cold acclimation on fitness in *D*. *melanogaster*. First, we confirmed that cold tolerance and the capacity to acclimate varies among naturally derived *D*. *melanogaster* genotypes, illustrating the beneficial effect of acclimation on survival. Next, we demonstrated that exposure to cold acclimation temperature decreases reproductive fitness, illustrating a negative fitness effect of acclimation when individuals are mismatched in stress exposure. Finally, we demonstrated that genetic capacity to increase survival through acclimation has little influence on the effect of acclimation on reproductive fitness, suggesting that the combined fitness effects of acclimation on survival and reproduction do not depend solely on genotype in environments subject to daily and seasonal thermal variation. Because populations of *D*. *melanogaster* are likely to experience mild cold stress (modeled by the acclimation treatment in our study) during the early morning hours of a spring or fall day, we suggest that the significantly decreased mating success of males exposed to the acclimation temperature will have biologically important consequences for fitness and the evolution of plasticity and cold tolerance in natural populations.

## Supporting information

S1 Fig *D*. *melanogaster* song. Oscillogram presenting an example of song produced by *D*. *melanogaster* males.
(TIF)

S2 Fig Estimation of the mean IPI using cut-offs. Each panel is labeled with the song ID, which corresponds to data available in S1 File.
(EPS)

S3 Fig Boxplots of variation in courtship duration (A) and courtship latency (B) measured in 19 DGRP lines. The male treatment (TRT) is indicated by shading with grey shading indicating the males that were exposed to the acclimation treatment (AC) and no shading indicating males that were not exposed (C).
(EPS)

S1 Table Sample sizes for each assay. (DOCX)

S1 File Raw data. (XLSX)

S2 File R code used in bootstrapping analysis. (TXT)
