# Supplementary figures and images for: Costs of cold acclimation on survival and reproductive behavior in Drosophila melanogaster
Source: PLoS One. 2018 May 23;13(5):e0197822. doi: 10.1371/journal.pone.0197822 (PMC5965859; doi:10.1371/journal.pone.0197822)

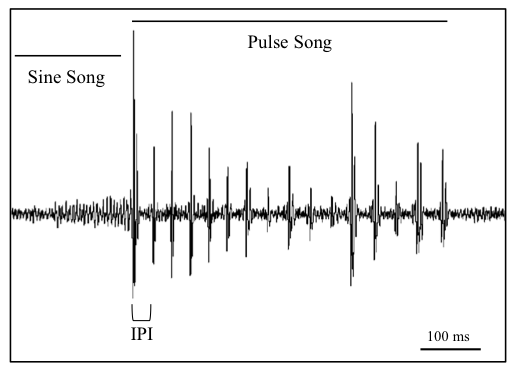

Supplement: S1 Fig — Oscillogram presenting an example of song produced by D. melanogaster males. (TIF) [file pone.0197822.s001.tif]
