# Supplementary material for: Costs of cold acclimation on survival and reproductive behavior in Drosophila melanogaster
Source: PLoS One. 2018 May 23;13(5):e0197822. doi: 10.1371/journal.pone.0197822 (PMC5965859; doi:10.1371/journal.pone.0197822)
Supplement: S1 Table — (DOCX) [file pone.0197822.s004.docx]

| DGRP Line | Cold Tolerance Assay  (per Sex) | Mating Assay  No. per Treatment (CC, CS, SC, SS*) | | Courtship Song Assay  (No. Acclimated, No. Control) |
| --- | --- | --- | --- | --- |
|  |  | Courtship Latency | Courtship Duration |  |
| RAL_304 | 4 | 6, 6, 6, 6 | 3, 4, 3, 4 |  |
| RAL_362 | 4 | 7, 7, 7, 7 | 2, 6, 6, 5 | 11, 16 |
| RAL_517 | 4 | 8, 8, 8, 8 | 2, 2, 3, 1 | 18, 14 |
| RAL_365 | 4 | 6, 6, 6, 6 | 4, 4, 2, 3 | 17, 16 |
| RAL_93 | 4 | 8, 8, 8, 8 | 7, 8, 3, 3 |  |
| RAL_101 | 4 | 7, 7, 7, 7 | 6, 5, 6, 6 |  |
| RAL_136 | 4 | 8, 8, 8, 8 | 4, 3, 2, 1 |  |
| RAL_153 | 4 | 5, 5, 5, 5 | 5, 5, 4, 4 | 16, 13 |
| RAL_176 | 4 | 8, 8, 8, 8 | 4, 4, 4, 1 |  |
| RAL_177 | 4 | 7, 7, 7, 7 | 6, 6, 4, 3 |  |
| RAL_195 | 4 | 8, 8, 8, 8 | 5, 5, 4, 4 | 16, 11 |
| RAL_336 | 4 | 8, 8, 8, 8 | 6, 6, 5, 6 |  |
| RAL_352 | 4 | 5, 5, 5, 5 | 2, 4, 5, 3 |  |
| RAL_359 | 4 | 8, 8, 8, 8 | 5, 4, 4, 0 |  |
| RAL_361 | 4 | 7, 8, 8, 8 | 2, 3, 3, 1 |  |
| RAL_367 | 4 | 5, 5, 5, 5 | 2, 2, 3, 4 |  |
| RAL_440 | 4 | 7, 7, 7, 7 | 4, 5, 5, 0 |  |
| RAL_849 | 4 | 7, 7, 7, 7 | 3, 6, 6, 6 |  |
| RAL_406 | 4 | 8, 7, 8, 8 | 3, 4, 3, 3 |  |
| *CC = Control male, Control female; CS = Control male, Acclimated female; SC = Acclimated male, Control Female; SS = Stressed male, Stressed female | | | | |
